# Deep learning with digital holographic microscopy discriminates apoptosis and necroptosis

**DOI:** 10.1038/s41420-021-00616-8

**Published:** 2021-09-02

**Authors:** Joost Verduijn, Louis Van der Meeren, Dmitri V. Krysko, André G. Skirtach

**Affiliations:** 1 Nano-Biotechnology Laboratory, Department of Biotechnology, Faculty of Bioscience Engineering, Ghent University, 9000 Ghent, Belgium; 2 Cancer Research Institute Ghent, 9000 Ghent, Belgium; 3 Cell Death Investigation and Therapy (CDIT) Laboratory, Anatomy an Embryology Unit, Department of Human Structure and Repair, Faculty of Medicine and Health Sciences, Ghent University, 9000 Ghent, Belgium; 4 Department of Pathophysiology, Sechenov First Moscow State Medical University (Sechenov University), 119146 Moscow, Russian Federation

**Keywords:** Cell death, Cellular imaging, Cancer imaging, High-throughput screening

## Abstract

Regulated cell death modalities such as apoptosis and necroptosis play an important role in regulating different cellular processes. Currently, regulated cell death is identified using the golden standard techniques such as fluorescence microscopy and flow cytometry. However, they require fluorescent labels, which are potentially phototoxic. Therefore, there is a need for the development of new label-free methods. In this work, we apply Digital Holographic Microscopy (DHM) coupled with a deep learning algorithm to distinguish between alive, apoptotic and necroptotic cells in murine cancer cells. This method is solely based on label-free quantitative phase images, where the phase delay of light by cells is quantified and is used to calculate their topography. We show that a combination of label-free DHM in a high-throughput set-up (~10,000 cells per condition) can discriminate between apoptosis, necroptosis and alive cells in the L929sAhFas cell line with a precision of over 85%. To the best of our knowledge, this is the first time deep learning in the form of convolutional neural networks is applied to distinguish—with a high accuracy—apoptosis and necroptosis and alive cancer cells from each other in a label-free manner. It is expected that the approach described here will have a profound impact on research in regulated cell death, biomedicine and the field of (cancer) cell biology in general.

## Introduction

Understanding of the molecular mechanisms of cell death is growing exponentially and our awareness of their key role in several disease models is steadily increasing [1]. Initially, cell death was divided into regulated and accidental cell death, where apoptosis describes the former [2], while necrosis represents the latter [3]. Apoptosis is characterized by membrane blebbing and caspase activation. However recently, this two way division was let go and many different regulated forms of necrosis, depending on the type of stimulus, that follow defined steps and signaling events were defined [1]. Necroptosis is an example of this regulated necrosis with the morphological features of cell rounding and swelling. It is mediated by the receptor-interacting protein kinase-1 (RIPK1), and its substrate, mixed lineage kinase domain-like (MLKL) [4, 5], and it has been implicated in several human pathologies such as chronic inflammatory diseases (e.g., inflammatory bowel disease and Chron’s disease), and neurodegenerative diseases (Alzheimer’s, Parkinson’s and amyotrophic lateral sclerosis) [6, 7]. It is important to distinguish apoptosis from necroptosis, particularly because necroptosis is often associated with inflammation under pathological conditions [4–6, 8].

It is essential to note that apoptosis and necroptosis are currently mainly analyzed by fluorescence microscopy or flow cytometry [9]. These techniques are costly, potentially phototoxic, and may even interfere with the cell death process itself [10–14]. In addition, flow cytometry is used to identify cell death types, however, it has been shown to not always be able to discriminate between apoptosis and necroptosis [9, 15]. Recently, atomic force microscopy (AFM) has been used to obtain information about the nanotopography and mechanobiology of dying cancer cells, without the use of labels, by probing them with a cantilever [16]. However, the AFM method depends on physical contact with the surface of adherent cells and has a low throughput. Therefore, there is a need to develop novel alternative approaches which will allow to study these cell death types and discriminate them from each other.

Digital Holographic Microscopy (DHM) is an approach solely based on visible light transmitted through a (dying) cell. In fact, this is a label-free technique based on phase shift microscopy, whereby there is a delay in light’s phase to visualize transparent cells. The DHM technique enables quantification of the phase shift of transmitted light. Additionally, this quantified phase image can be converted into the topography of cells [17–20]. DHM has already been used to identify cell death after l-glutamate exposure [20] and to distinguish between apoptosis and oncosis (i.e., accidental necrosis) [19]. However, the full potential of DHM coupled with convolutional neural network data analysis has—to the best of our knowledge—not yet been exploited. Therefore, we incorporated an automated data analysis to increase applicability and throughput of DHM. In parallel to developments in the field of microscopy, image processing is experiencing an explosive growth, sparked by the application of artificial intelligence in many biomedical applications [21, 22]. Therefore, we have applied a deep learning model to microscopic image processing, enabling label-free microscopy approaches [23].

Artificial intelligence (such as deep learning) has already been applied to label-free microscopy techniques to, for example, classify cancer cells [24, 25] and more specifically skin cancers [26]. More recently, this combination of classifying cells and label-free microscopy has been applied on regulated cell death research [27, 28]. However, both of these label-free cell death investigations do not exploit the potential of the predictive power of deep learning, more specifically, the use of convolutional neural networks.

Herewith, we apply deep learning algorithms, coupled with label-free DHM, to distinguish between alive, apoptotic and necroptotic cancer cells, with a high accuracy. This new approach is expected to have a profound impact on cell death research, biology and even medicine because this enables to identify cell morphology and cell fate in a label-free manner. This will lead to the incorporation of label-free analysis tools into current standards in scientific research such as flow cytometry, complementing and eventually replacing the need for fluorescent labels all together. A combination of a label-free detection method DHM with deep learning, will allow picking up on small features within large cell populations. Eventually, this will lead to finding label-free features to detect, for example, early stages of cell death and different cell death modalities, which are directly linked with immunogenic cell death and immunotherapy.

## Results

### Characterization of apoptotic versus necroptotic cancer cell death

**Fig. 1:**
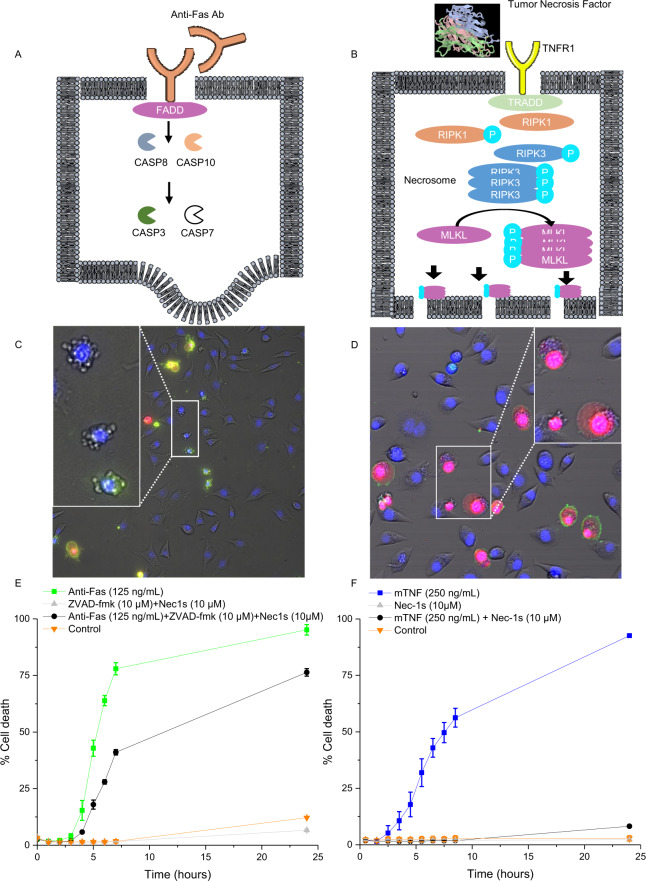
Induction of apoptosis and necroptosis in L929sAhFas cells by anti-Fas antibodies and mTNF, respectively. **A** An overview of extrinsic apoptosis is shown schematically, where the added anti-Fas antibody binds to the anti-Fas receptor, activating Fas associated death domain (FADD). This in turn activates the caspase cascade. Starting at caspase 8 and 10 activation and resulting in activation of caspase 3 and 7 and in turn apoptosis. **B** An overview of induction of necroptosis by mTNF which binds to the mTNF receptor 1 and activates the intracellular TNF receptor associated death domain (TRADD). Activation of TRADD results in activation of RIPK1 and RIPK3, with the latter being responsible for the formation of the necrosome. The necrosome in turn phosphorylates MLKL (mixed lineage kinase domain like pseudokinase) to create a pore forming complex in the membrane and thus leading to necroptotic cell death. **C**, **D** Induction of regulated cell death in L929sAhFas cells using anti-Fas and mTNF analyzed by fluorescence microscopy. A representative fluorescent image of L929sAhFas cells induced to die by apoptosis (**C**, 125 ng/mL anti-Fas) or necroptosis (**D**, 250 ng/mL mTNF). The following fluorescence stainings are used in these images: Hoechst 33342 (nuclei), propidium iodide (PI, staining of the nuclei after cell membrane rupture) and annexin-V (phosphatidylserine (PS) exposure). PS exposure happens in early apoptosis and can be externalized in necroptosis. Thus cannot be used to differentiate between cell death types 47. Zooms of both conditions clearly show regulated cell death specific features, as membrane blebbing in apoptosis and swelling of the cell in necroptosis. **E**, **F** A quantification of cell death by fluorescent microscopy over time. Images were obtained after induction of each experiment and relative PI exposure (and thus percentage of cell death) was measured over time. Clearly the induction of both apoptosis and necroptosis (using anti-Fas and mTNF, respectively) work efficiently. The inhibitors for both apoptosis (10 μM of zVAD-fmk, pan-caspase inhibitor) as necroptosis (10 μM of Nec-1s) have been used. Blocking caspase activity using zVAD-fmk, after induction of apoptosis using anti-Fas, will lead to a switch to necroptosis and thus an increase in cell death 38,39 which can be further blocked by Nec-1s.

In this work, we have used the murine L929sAhFas fibrosarcoma cell line, a well-known cellular model, to induce two different regulated cell death modalities in one cellular context: apoptosis and necroptosis (Fig. 1A, B respectively). By adding anti-Fas antibody, the extrinsic apoptosis pathway is triggered via the hFas receptor [29], while necroptosis is induced by mTNF [30, 31]. By using fluorescence microscopy (Fig. 1C, D), it is demonstrated that the induction of both apoptosis and necroptosis was done successfully. Morphological features of both apoptosis and necroptosis are observed (Fig. 1C, D). In order to assess apoptotic versus necroptotic cell death, several cell death inhibitors have been applied [32]. A pan-caspase blocker zVAD-fmk was used to block caspase activity in anti-Fas induced apoptosis, however blocking the caspase activity can lead to a switch from apoptosis to necroptosis. This is why in these experiments zVAD-fmk as well as Nec-1s was applied to suppress cell death (Fig. 1E). To inhibit mTNF induced necroptosis, a RIPK-1 inhibitor Nec-1s was used (Fig. 1F) which efficiently reduced cell death rate. All these data confirmed that anti-Fas and mTNF specifically induced apoptosis and necroptosis, respectively, in L929sAhFas cells confirming the previously published data [33].

### Experimental setup and imaging using DHM

**Fig. 2:**
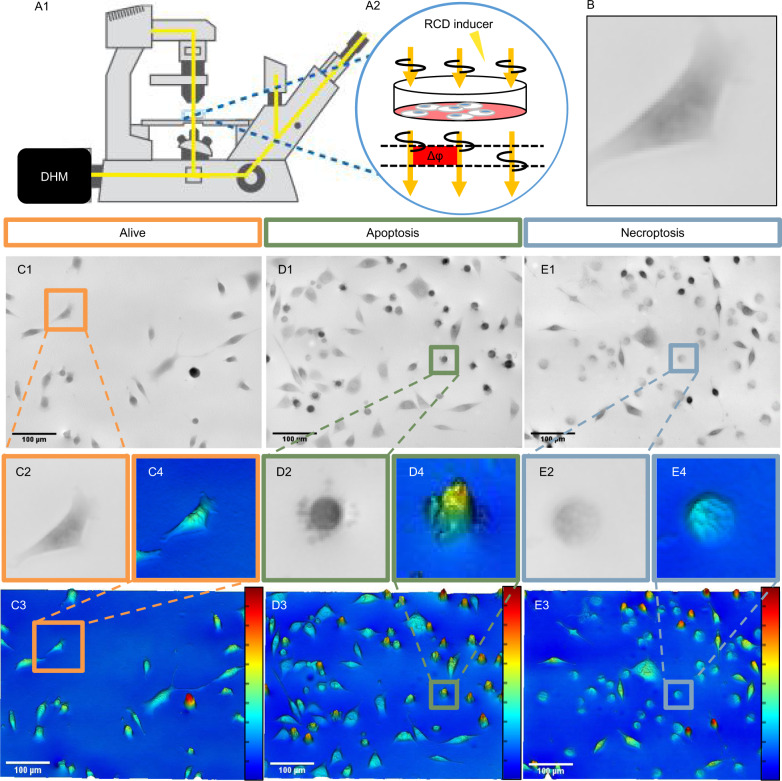
The application of digital holographic microscopy on regulated cell death. **A1** A schematic representation of the DHM setup. 5.5 ×104 cells/mL of L929sAhFas cells were grown in a glass bottom dish with a 15 mm glass coverslip and, depending on the experiment a cell death inducer was added. **A2** Transmission light was applied in an inverted microscope setup. Light beams that interfered with cells resulted in a phase delay, which is quantified by the DHM camera and saved as a hologram. **B** An example of images used as input for the deep learning neural network. **C1**–**C4** Hologram images of alive cells (without any cell death inducer). C1 is the phase image of the complete field of view, with C2 being a single cropped image from this capture. Images similar to C2 were used as input towards model development shown in Fig. 3B. C3 and C4 are 3D representations of the optical height from the holograms, with a relative scale bar (100 µm). C2 and C4 represent the same cell. **D1**–**D4** A similar layout as given for C1–C4, however, cells are induced either to undergo apoptosis with 125 ng/mL of anti-Fas antibody. For **E1**–**E4** the cells were exposed to 100 ng/mL of mTNF to induce necroptosis.

After the induction of cell death as described earlier, phase contrast images were obtained in a DHM setup (Fig. 2A). It uses a conventional optical light microscope with a camera producing digital holographic microscopy images (Fig. 2B). Such an approach allows for acquisition of DHM images without the need for fluorescent markers and thus without a subsequent fluorescent setup. The holographic microscopy images are obtained straightforwardly since only a transmission microscope is needed. Additionally, the DHM images provide quantitative information about morphology and height profiles (Fig. 2C1–E4). In order to confirm the data obtained through the DHM setup, we have quantified the induction of apoptosis and necroptosis by an established fluorescence microscopy assay based on the markers Hoechst 33342 and PI which allow to confirm the occurrence of cell death and to obtain total cell counts (Supplementary Fig. 1). One can observe on DHM phase contrast images typical features for apoptotic cells, namely blebbing and cell shrinkage (Fig. 2D1–D4) and typical characteristics for necroptosis such as rounding up and swelling of the cells (Fig. 2E1–E4).

### Transforming the DHM images to input for the deep learning algorithm

DHM images were used for subsequent analysis using a deep learning approach. In order to obtain captures (an image over the complete field of view), experiments were performed in cells that were not treated with any inducer or cells induced with either an apoptotic or necroptotic cell inducer. Only one type of cell death inducer per experiment was used and cells were imaged as explained above (Fig. 2C1–E4). For deep learning, single cells were cropped from each capture and inserted into the deep learning algorithm (Fig. 2C2, D2, E2).

### Overcoming class mislabeling using supervised anomaly detection

**Fig. 3:**
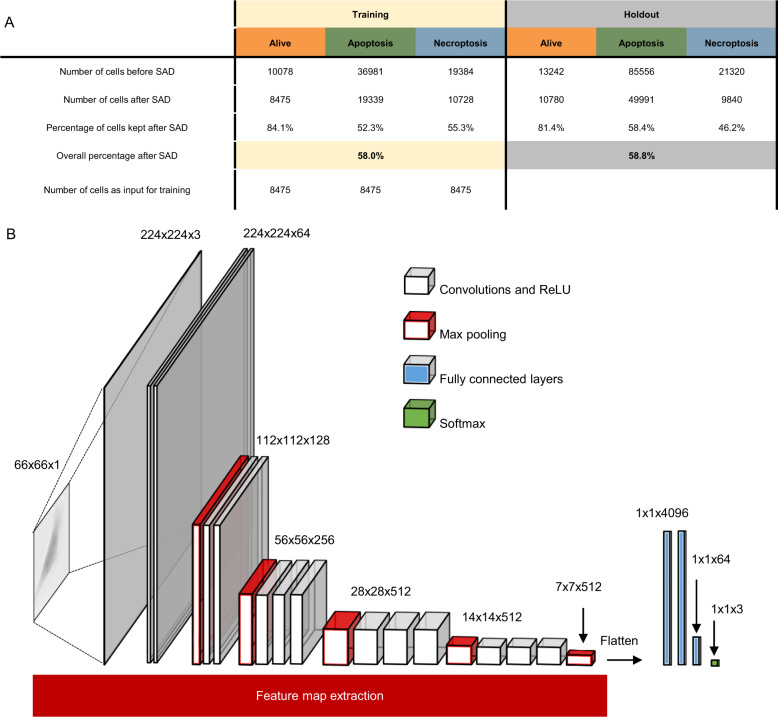
The analysis pipeline, supervised anomaly detection followed by deep learning using VGG-19. **A** The number of cells in each category before and after filtering with supervised anomaly detection (SAD). On the left the numbers of cells in the training data (subdivided into the three categories) and on the right this for the holdout dataset (a set of data with independent experiments). This shows that 58% percent of cells are kept after SAD. Afterwards, the training data is trimmed to overcome unequal amounts of input and thus circumvent unbalanced training data, this is equal to the smallest category of the training set. This trimmed dataset is used for the learning process as seen in Fig. 4. **B** A schematic representation of VGG-19 with several steps of convolution, activation and maximal pooling from left to right. This shows several steps within the convolutional neural network, starting from an image (left). Here, the cropped cells are used with the size of 66 × 66 pixels, these are resized to 224 by 244 over three channels to match the input size of the VGG-19 groundworks. After resizing the input, there are several blocks of convolution to extract information from these images. This is done by applying a convolution matrix on top of the original image which takes into account the values of surrounding pixels to recalculate and accentuate the image. Followed by activation of a rectified linear unit (ReLU), this will eliminate negative values after convolution. Max pooling, uses a 2 × 2 matrix to find and select the highest of these four values and thus reduces the size of the image stack by two in both width as height. This can be seen in the image as well at every max pooling step (indicated in red), the height and width are divided by two and the depth is multiplied by two. This creates different sizes of image stacks moving further into the feature map extraction. In this model these two combinations (convolutions + ReLU and max pooling) always follow up on each other. This is done to non-linearize the outputs of each convolution and to optimize the predictive value of the model. After all the convolution steps, the image stack (at the end of the convolutions of size 7x7x512), is flattened to be used in the following step of the model and neural network part of the Convolutional Neural Network: the fully connected layers. Nodes from each layer are connected to all nodes of the next layer of this neural network. Each node gets input from the previous layer and will provide an output towards the next layer of the network. Lastly, the softmax layer is applied, which is able to convert the neural activations into probabilities per category, or in other words, a prediction.

Cell death induction is a heterogeneous process because it affects each cell at different time points. Furthermore, it has a particular efficiency depending on the concentration of the inducer, the intracellular state and cell density. To overcome this, a supervised anomaly detection method (SAD) was applied. This process eliminates the alive cells from being labeled apoptotic or necroptotic and thus removes a percentage of cells from the population. A comparison between fluorescence (percentage of PI positive cells) and the SAD filter is shown in Supplemental Fig. 1. Both fitted curves (fluorescence and SAD filter outcome), show an increase in percentage of dead cells when the time after induction increases. Afterwards, the dataset was split into a training set and a holdout set in order to be able to train a model and, using independent experiments, verify the results. For both the training set and the holdout set 58% of the cells were kept (Fig. 3A).

### Transfer learning for the discrimination of apoptosis and necroptosis

To predict between apoptosis and necroptosis and the control, i.e., alive L929sAhFas cells, an existing model, the VGG-19 network [34], was used as the starting point to further develop and fine-tune towards the goal of differentiation. VGG-19 consists of several repeating building blocks structured in a slightly different manner (Fig. 3B). The first step is convolution, using a 3 × 3 convolution matrix. The image, a matrix of gray values, is passed through a stack of convolution matrices. The resulting stack with the same height and width is passed through a rectified linear unit (ReLU) which removes negative values from the stack with calculated pixel values. Of note, in this model these two steps are combined in order to non-linearize the repeating convolutions. After several rounds of convolution and ReLU’s, the resulting stack of matrices is condensed using maximal pooling. For this, a matrix of 2 × 2 is applied to the image stack, taking the maximum value from these four pixels thus reducing the width and height of the stack by 2 (Fig. 3B in red). This whole process of convolution, activation and pooling steps (Fig. 3B, white and red blocks) is called feature map extraction. It creates a map with all features of the image, which will be used later in the fully connected layers (Fig. 3B in blue). The output of convoluting and pooling the stacks of images, is flattened into a single vector (Fig. 3B in blue). This vector is then used as an input for the fully connected neural network part of the model. Within this neural network, all nodes are connected with all nodes from the previous and next layer, the importance of each connection is stored in a weight factor. Each node takes its input signal and associated weight to generate a weighted output. The last layer is the output layer containing, in this case, three categories: alive, apoptosis and necroptosis (Fig. 3B, in green).

**Fig. 4:**
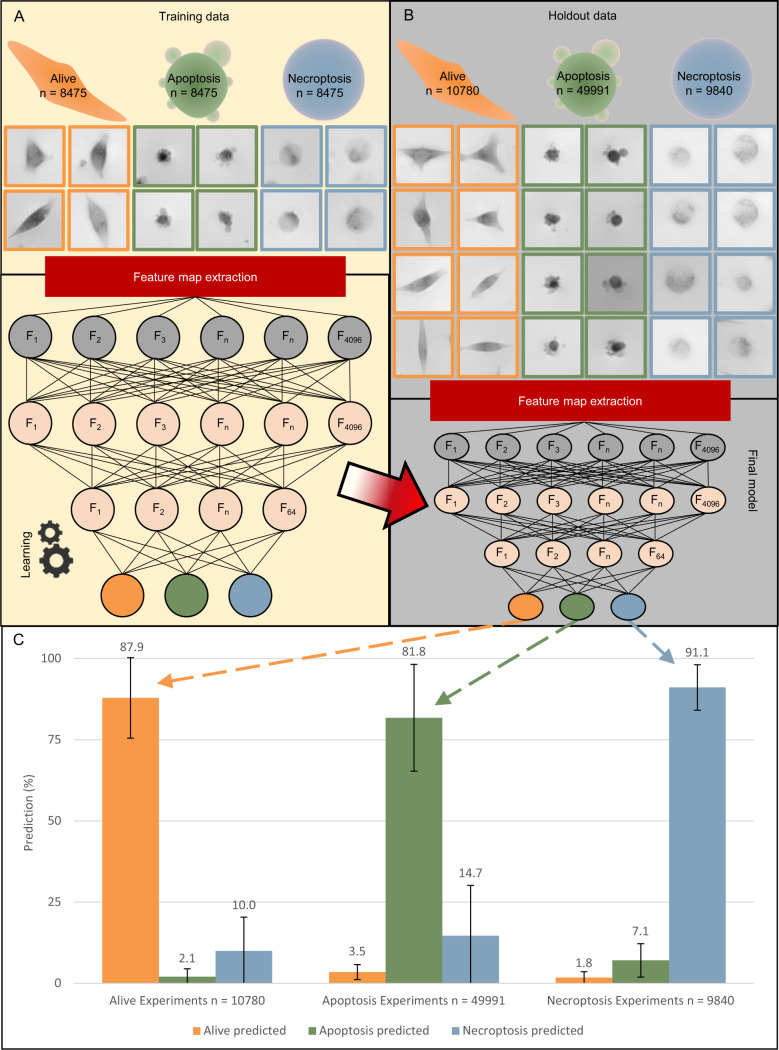
The creation of the deep learning algorithm by transfer learning from VGG-19. **A** The first step of the model creation; training of the model. The test data is inserted with both label and image, here the algorithm is fine-tuned towards the goal of differentiating between alive, apoptosis and necroptosis. After the model is created, the holdout data set is inserted into the model, as seen in **B**. **B** Holdout data is inserted into the model, without identifying the label to the model. Afterwards, the label of the image is compared with the prediction outcome of the model. this is displayed as an accuracy percentage by dividing the correct number of predictions by the total amount of cells per input label in **C**. **C** Accuracy percentages are displayed as bar charts showing that 87.9%, 81.8% and 91.1% of cells in alive, apoptotic and necroptotic experiments respectively are predicted correctly.

**Fig. 5:**
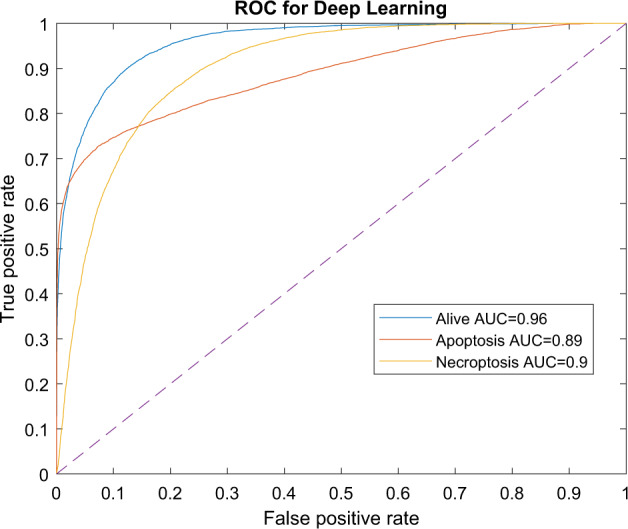
Area under the curve represents a comparison between three categories (alive, apoptosis and necroptosis). Receiver operating characteristic (ROC) plot is shown with the true positive rate (or sensitivity) on the *y*-axis and the false positive rate on the *x*-axis (or specificity). The diagonal line in the middle indicates a random guess with an AUC of 0.50. From this graph, the classification performance of the three categories, are compared. The final model functions best when discriminating alive cells (AUC = 0.96) compared to apoptosis and necroptosis (AUC 0.89 and 0.90 respectively). However, all three classifiers function noticeably better than the random guess at an AUC of 0.50.

The data was split in two subsets: training (Fig. 4A) and holdout data (Fig. 4B). The training set (~35,000 cells) was used to tune the model towards its new objective of detecting and discriminating between alive, apoptotic and necroptotic cancer cells. This training set was in turn split into training (80% from the training set ~28,000 cells) and test (20% of original training set, ~7000 cells) sets. The training set was used to fine tune hyper parameters of the model, whilst the test set was used to provide an unbiased intermediate evaluation of the model. The holdout set (~70,000 cells), containing cells of independent experiments, was used to predict and validate the accuracy of the model itself (Fig. 4B, C). Resulting in 88%, 82 and 91% accuracy on the holdout set for alive, apoptotic and necroptotic cells respectively (Fig. 4C). The accuracy is calculated by the number of cells predicted to the correct category divided by the total number of cells in the associated experiments (Fig. 4C). The receiver operating characteristic (ROC curve), is a plot showing the true positive rate compared to the false negative rate (Fig. 5). The area under each curve, gives an indication of the overall performance of each classifier with a higher area under each curve being a better classifier. The diagonal dashed line in the middle gives the area under each curve of a random guess. The alive classifier (area under each curve of 0.974) outperforms the classifiers for apoptosis and necroptosis (area under each curve 0.923 and 0.924, respectively). However, they all are better than the random guess with an area under the curve of 0.500. A random guess will result in 50% correct predictions and 50% incorrect predictions, when plotted this will give the dotted diagonal line.

## Discussion

### Molecular mechanism of regulated cell death

As the first step, we demonstrated that the induction of both apoptosis and necroptosis was done successfully by using fluorescence microscopy (Fig. 1C, D), currently the most widely used approach for distinguishing between different cell death modalities. Typical morphological features were present in apoptosis, including membrane blebbing and cell shrinkage whereas annexin-V staining indicated the stage of apoptosis (Fig. 1C as green). In necroptosis the increasing of cell volume, typical membrane protrusion and rupture of the plasma membrane integrity was visualized by propidium iodide (PI) positivity (Fig. 1D in red). Apoptosis, which can be induced by anti-Fas antibodies, can switch to necroptosis due do the caspase inactivation by zVAD-fmk [35, 36]. This is why Nec-1s was added in addition to zVAD-fmk, to block both the anti-Fas induced apoptosis (by zVAD-fmk) and inhibit the cells that switched to necroptosis (by Nec-1s) (Fig. 1E). For mTNF induced necroptosis, it can be inhibited by adding Nec-1s (Fig. 1F). These results indicate that stimulation of L929sAhFas cells with anti-Fas antibodies and mTNF leads to induction of apoptosis and necroptosis, respectively.

### Digital holographic microscopy experimental setup

The DHM setup (Fig. 2A) is straightforward to design, because it only uses a regular transmission microscope which enables detecting its phase delay using normal transmission light (Fig. 2A2). Light passing through cells will be delayed in its phase and this phase delay can be quantified using the DHM setup. Equation 1 adopted from Pavillion, shows the relation between the phase delay and height (*h*) of a sample [20]:

1 $${\Delta}\varphi = \frac{{2\pi }}{\lambda }\left( {\eta _i - \eta _{media}} \right) \ast h$$

where *Δφ* is the phase delay (Fig. 2A2), *λ* is the wavelength of light, *η*_*i*_ is the mean refractive index of cells, *η*_*media*_ is the refractive index of the media and *h* the height of the cells. The phase delay of the light propagating through a sample depends on its height and its refractive index [20], since the wavelength (*λ*) and the refractive index of the medium (*η*_*media*_) are fixed parameters. The mean refractive index of the cell (*η*_*i*_) can be set to a fixed value by comparing cells with and without media [37]. The resulting height calculated from the phase delay is referred to as ‘optical height’. This conversion between the phase delay in the quantitative phase images (Fig. 2C1, D1, E1), and the 3D profile (Fig. 2C3, D3, E3), can be seen in these figures where the color scale at the right-hand side of the 3D image is relative to the height of the corresponding cells. It can be seen in Figure 2 that the difference in morphology between the three samples (alive, apoptosis and necroptosis) is visible in both the quantitative phase images (Fig. 2C1, D1, E1) and the 3D optical height images (Fig. 2C3, D3, E3). These height profile images, especially the zooms (Fig. 2C4, D4, E4), show detailed cellular morphology. There is an increase in optical height in apoptosis (Fig. 2D4), this could be due to the chromatin condensation (one of the classical hallmark of apoptosis). This increases the refractive index of the cell, because there is a change in biochemical composition, leading to an increased optical height. Additionally, in necroptosis (Fig. 2E4) a decrease of optical height is observed, this is likely due to pore formation in the cell membrane, leading to a smaller difference in refractive index between the cell and the medium. This in turn lowers the perceived optical height.

### Deep learning analysis

The heterogeneity in cell death response (after induction) is a hurdle to overcome when incorporating data into further predictive models. Thus, the heterogeneity of cell populations necessitated a pre-filtering of cells before entering the deep learning model. In our work, this pre-filtering is done by a supervised anomaly detection (SAD). For this, SAD was used to remove the clearly living cells from experiments in which a cell death inducer was applied [38]. This model was provided with 200 images of alive cells and 100 images of both apoptotic and 100 images of necroptotic cells. It can be emphasized that this SAD-model was capable of determining whether input samples were more alive- or more death-like. Subsequently, the outcome of this SAD-model was used to pre-filter the datasets. By doing so, cells populations used as input for the model development were purified to only contain cells of a particular type. The effectivity of the SAD model is compared to a fluorescence based technique (Supplemental Fig. 1), both the SAD filter as the fluorescence show an increase of dead cells over time.

Moreover, we have used a transfer learning approach [39], in which an existing model, VGG-19 [34], was reworked to predict and differentiate between three groups of cells: alive, apoptotic and necroptotic cells (Fig. 3B). This model is a convolutional neural network, i.e. it uses convolutions to extract important features from an image (white and red boxes Fig. 3B), coupled with a fully connected neural network to produce a prediction (blue and green boxes in Fig. 3B) [40].

To avoid potential phototoxicity of fluorescent markers, we have explored the capabilities of DHM, combined with deep learning as a label-free detection method based on quantifying the phase delay created by transparent objects, e.g., cancer cells. In our work, we describe that a convolutional neural network is an excellent approach for processing biological images in prediction purposes, since it does not rely on a pipeline to extract features from the images [26]. Peculiarly, this is in contrast to classical machine learning applications [39]. With an appropriate analysis pipeline, the potential of deep learning can be applied. Where, first of all, the cells are extracted from the captures (Fig. 2B), followed by pre-filtering using a SAD (Fig. 3A) and, lastly, a model is trained to classify the cells accordingly (Fig. 4A). Once the training is finalized, the holdout data set is applied on the constructed model, in order to validate it (Fig. 4B). The presented approach yields a reliable method for prediction of regulated cell death with a remarkable accuracy of 87.9%, 81.8% and 91.1% for alive, apoptotic and necroptotic cells, respectively (Fig. 4C). More importantly, this prediction is done without using fluorescent labels. We envision that this model has the potential to lead towards the creation of an even more refined model capable of distinguishing a whole range of regulated cell deaths or other cell morphologies.

To validate the model in more depth than just the accuracy rates, we have investigated the receiver operator curve (ROC), as it can be seen in Fig. 5. In a ROC graph one looks at the true positive rate versus the false positive rate. Each curve is a binary evaluation for each category of the model, where one looks into the likelihood of the model to be correct for each group. A perfect test will give an area under the curve of 1, a random test 0.5 [41]. For example, the area under the curve for the necroptosis classifier is 0.924. This means that a random cell from the necroptosis experiments has a higher prediction score for this classifier than a random cells from any other experiments 92.4% of the time [42, 43].

The current label-free cell death investigations do not exploit the potential of the predictive power of deep learning [27, 28]. Barker and colleagues use a decision tree to make their classification after extraction of parameters from the images [28]. Vicar and colleagues use a support vector machine classifier based on a combination of extracted features named ‘cell dynamic score’ and normalized density [27]. Using a convolutional neural network as done in the current research produces a powerful approach for cell death prediction and later on can be applied to other problems with a difference in cellular morphology.

### Outlook

In the future we think that applications where deep learning is used in combination with label-free microscopy techniques will add new information regarding cell subtyping. By the addition of these new parameters and analysis techniques, information can be added on top of fluorescence microscopy. Additionally the deep learning algorithm described here can be further developed into other applications within biology, e.g., automated drug screening.

## Conclusions

In conclusion, we have demonstrated a new label-free DHM based method to predict and distinguish between regulated cancer cell death modalities, namely apoptosis and necroptosis, at high throughput rate. DHM has been coupled with deep learning algorithms to process information and make a formidable prediction with >85% of correctly identifying each cell death modality (alive, apoptosis or necroptosis). The deep learning model is based on convolutions that are capable of extracting features from images. This model is envisioned to find applications in research on cell death by facilitating non-fluorescence methods, e.g., drug screening in an automated manner [44]. In this way, it can be indicated that regulated cell death modality is activated upon induction with an unknown compound.

## Material and methods

### Cell culture

L929sAhFAS murine fibrosarcoma cells [29, 33, 45, 46] were cultured using a high glucose (4.5 g/L) Dulbacco’s Modified Eagle’s medium (DMEM) (LONZA, 12-604 F). Media were supplemented with 10% fetal bovine serum (FBS) (ThermoScientific, 10500064), 100U Penicillin and Streptomycin per mL (Lonza, DE17-602E). Cells were cultured at 37 °C and 5% CO_2_ atmosphere. Cells were kept under 80% confluency and detached using Trypsin (Lonza, 17-161E). Cell line is tested negative for mycoplasma using a PCR mycoplasma kit (PromoKine, PK-CA91-1096). L929 cells were selected for mTNF sensitivity and transfected with human FAS receptor [29, 45, 46].

### Induction of apoptosis and necroptosis

For the induction of regulated cell death, different products were used. By adding 125 ng/mL anti-Fas antibody (Merck, 05-201) the external apoptosis pathway was triggered via the FAS receptor (Fig. 1A). Murine Tumor Necrosis Factor (mTNF) was used in a concentration of 125 ng/μL to stimulate necroptosis (Fig. 1B) (recombinant protein from VIB Protein Core Facility).

### Fluorescence microscopy setup

Fluorescence microscopy was used to verify the cell death. Cells were seeded on a confocal dish (VWR, 734-2903) one day prior to imaging and incubated at 37 °C and 5% CO_2_. The acquisition took place on a Nikon Ti microscope equipped with blue, green, and red emission channels. Cells were analyzed using propidium iodide (PI) and Hoechst 33342 at a concentration of 1 μg/mL (Thermofisher, P1304MP and Thermofisher, 62249). Hoechst 33342 was used to stain all nuclei and PI to detect all dead cells (cell membrane permeabilization) followed by capture of a 7000 μm by 7000 μm area. Using these images, the percentage of PI ^+ ^cells was calculated.

### Digital holographic microscopy experimental setup

Cells were seeded on a confocal dish (VWR, 734-2903) one day prior to imaging and incubated at 37 °C and 5% CO_2_. Cells were placed inside the microscope. Whilst inside, the incubation chamber of the microscope, inducer and fluorophores (Hoechst 33342 and PI) were added to the dish and subsequently imaged. The acquisition took place on a Nikon Ti microscope.

### Imaging setup

Images obtained in this work were made using the Nikon Ti, equipped with a digital holographic camera Qmod (Ovizio, Belgium). Both temperature and CO_2_ (37 °C and 5% respectively) were controlled by an incubation chamber present at the microscope.

### Supervised anomaly detection

To filter all cells that showed alive morphology from experiments where inducers were used, a supervised anomaly detection (SAD) filter was applied. This algorithm looks at samples and detects whether cells are alive or dead. In order to make this filter, 200 alive cells, as well as 100 cells with apoptotic morphology and 100 cells with necroptotic morphology were selected by hand. These were placed in folders ‘Alive’ and ‘Death’. Next, AlexNet [47] was used to make a feature map from the above described supervised dataset. On this feature map, a supporting vector machine (SVM) was applied to make a characterization based on the feature map and the corresponding input labels. This filter is later on used to crop cells from the input captures (Fig. 2C2, D2, E2). Cells cropped from the captures that did not coordinate with their respective experiments were discarded. For example, in an experiment, where anti-Fas was added (and thus apoptosis was induced) all cells labeled ‘Alive’ were discarded.

### Application of SAD and cropping cells from capture

To locate cells within each capture, a script was used where cells were identified by thresholding. From the center of these objects, a box of 66 by 66 pixels is created to crop out the cells as long as they do not cross the border of the image and have a reasonable overall area (>80 px and <600 px). After cropping from the captures, the cells were inserted in the above mentioned SAD. The SAD outputs both the predicted class (‘Alive’ and ‘Death’) and an associated score. A threshold was applied to this prediction score. The higher the threshold, the more samples are excluded. However, lowering the threshold level will increase the data set and will give a broader range of samples for later use in the prediction model. If the prediction of the SAD aligned with the experiment and the prediction score was higher than the threshold (*T* = 0.01), the box was cropped and saved separately for use in the deep learning model.

### Model training and application

Creating an accurate prediction depends on the quality and quantity of the training data set, since this will affect the effectivity of the model. Since each object within an image represented a cell; our training data contained—after SAD—38,542 cells distributed over the three groups with 8475 alive cells, 19 339 apoptotic cells and 10 728 necroptotic cells (Fig. 3A). To counter unbalanced classes in the training data, the training set was cropped to the smallest set at random leading to 8475 cells in all three groups (Fig. 3A). This was then randomly split by class to an 80%-20% distribution, in which 80% of the data set is used to train the model and 20% of the training set is used to validate whilst training, to monitor overfitting and to give a better representation of its performance. Transfer learning was used [39] by repurposing an existing model. Here, we repurposed VGG-19 [34] by deleting the last three layers of the model and adding five layers at the end of the fully connected part of the model. To make this model applicable for our purpose, we added a fully connected layer of size 64, ReLU, fully connected layer of size 3 (amount of classes), followed by softmax and classification layers. After training the prediction model, the model was tested using a holdout set. This consisted of independent experiments that the model had never encountered before.

## Supplementary information

Supplemental Figure 1

Caption Supplemental Figure 1

## Data Availability

All data, models and MATLAB code are available in the Mendeley Data repository, 10.17632/sv5m953vjj.1.
