# Supplementary figures and images for: Deep learning with digital holographic microscopy discriminates apoptosis and necroptosis
Source: Cell Death Discov. 2021 Sep 2;7:229. doi: 10.1038/s41420-021-00616-8 (PMC8413278; doi:10.1038/s41420-021-00616-8)

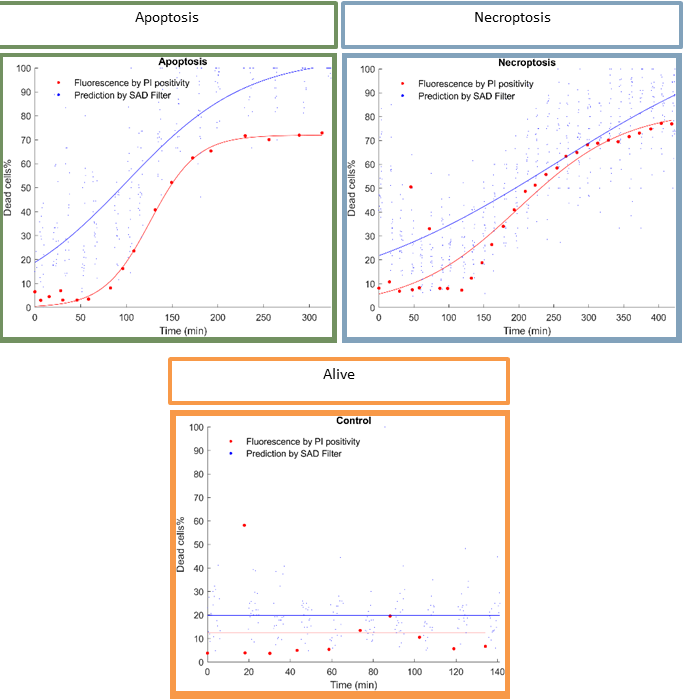

Supplement: Supplementary file 1 — Supplemental Figure 1 [file 41420_2021_616_MOESM1_ESM.png]
