# Supplementary material for: Deep learning with digital holographic microscopy discriminates apoptosis and necroptosis
Source: Cell Death Discov. 2021 Sep 2;7:229. doi: 10.1038/s41420-021-00616-8 (PMC8413278; doi:10.1038/s41420-021-00616-8)
Supplement: Supplementary file 2 — Caption Supplemental Figure 1 [file 41420_2021_616_MOESM2_ESM.docx]

Supplemental Figure 1. Comparison between fluorescence and the SAD filter in three different RCD modalities (Apoptosis, Necroptosis and Alive). In blue, the cell death prediction per capture is displayed, with a fit over these data indicated by the blue line. The red points are the percentage of PI positive cells ((number of PI positive nuclei/total number of nuclei by Hoechst 33342 stain)*100), with the red line being the fit through these points. All fits here are logistic fits with three parameters. On the x-axis the time post induction with appropriate inducer is displayed. Most noticeable is that the curve representing the output on the SAD filter (blue) and the PI positivity curve (red), are similar. Both trends increase when the time after induction increases.
